# Coral reef rehabilitation following Hurricane Irma using nano-engineered artificial reefs in Sint Maarten

**DOI:** 10.7717/peerj.18487

**Published:** 2024-12-17

**Authors:** Emily Higgins, Kyralai Duppel, Megan Connell, Guyon Brenna, Konstantin Sobolev

**Affiliations:** 1 IntelliReefs CAN, Halifax, Nova Scotia, Canada; 2 IntelliReefs, Salt Lake City, Utah, United States; 3 College of Engineering & Applied Science, University of Wisconsin, Milwaukee, Wisconsin, United States

**Keywords:** Coral reefs, Corals, Artificial reefs, Nanotechnology, Oceanite, Sint Maarten, Reef restoration, Ecosystem restoration

## Abstract

Artificial reefs are being increasingly deployed as a coral reef restoration strategy. Additional reef habitats made from conventional substrates (*e.g*., metal, concrete, *etc*.) have had limited success in addressing conservation objectives on degraded coral reefs due to structure size and lack of standardized monitoring, and inability to enhance select ecological, and species variables. Technological advances and new restoration methods must be quickly tested and applied on a large scale to curb further deterioration of coral reefs. Here, we present the results of the first deployment of Oceanite artificial reefs (ARs). We compare the composition of the benthic community and associated fish assemblages on Oceanite ARs 14 months after deployment in a marine protected area (MPA) and two unprotected sites in Philipsburg, Sint Maarten. We also examined fish abundance and behaviour on the ARs. The initial results from this pilot study suggest that Oceanite mineral matrices can enhance local biodiversity, attract coral recruits, provide food and protection for large fish communities, and develop an early stage, healthy coral reef community in 14 months. We suggest that further research and testing of Oceanite capabilities will allow us to develop site-, species-, and function-specific nanotechnology-enabled substrates to optimize AR conservation goals. Oceanite mix designs can be tuned to precise parameters to promote reef restoration and stressor mitigation (*e.g*., pH, leachate emissions, surface texture, porosity, void structure, and hydrophobic, heat-absorbing, and disease-fighting properties). Using both bottom-up and top-down restoration processes, we suggest that deploying bio-enhancing habitats with targeted microclimate stressor treatments on the world’s critical reefs will allow to build global refuges resilient to climate change and provide much needed ecosystem services.

## Introduction

Over the past four decades, coral reefs have experienced accelerated degradation due to multiple synergistic natural and anthropogenic stressors (Heron et al., 2016; Hughes et al., 2017). Rising ocean temperatures and acidification, overfishing, invasive species, and marine pathogens are contributing to a global loss in coral cover, structural complexity, and biodiversity (Hoegh-Guldberg et al., 2007; Hughes et al., 2003). As a result, there has been a rise in active or manipulative coral restoration projects in an attempt to mitigate ongoing deterioration (Rinkevich, 2015; Abelson, 2006). Artificial reefs are a targeted restoration method that aim to restore select ecosystem metrics (*e.g*., increasing coral cover and fish biomass, enhancing structural complexity on degraded reefs, *etc*.) (Higgins, 2019). Furthermore, artificial reefs have the potential to restore socio-economic and cultural benefits created by natural reefs, such as increasing local tourism, stabilizing food security, and providing environmental education (Chen et al., 2013).

Artificial reef (AR) deployment rates have increased over the past two decades (Higgins, 2019) and are primarily integrated within natural coral reef systems to add or enhance colonizable substrate for benthic organisms and to create habitat and shelter for motile fish and invertebrate species (Carr & Hixon, 1997; Baine, 2001; Perkol-Finkel, Shashar & Benayahu, 2006; Barott & Rohwer, 2012). Additionally, ~40% of ARs are submerged specifically to remediate damage to commercially important target populations (Abelson, 2006; Higgins, 2019). The success of AR projects in achieving these deployment objectives is highly dependent on chronic and acute local environmental stressors, design and scale of the structure, integration into existing conservation landscape, marine spatial planning, and adaptive management by local stakeholder groups (Higgins, 2019; Boström-Einarsson et al., 2020). ARs also have mixed success globally due to background disturbances, such as bleaching and acidification, and are rarely produced at an ecologically relevant scale; many ARs and man-made underwater structures designed for reef restoration are made from materials of opportunity that prioritize price over engineering effort (Perkol-Finkel, Shashar & Benayahu, 2006; Higgins, 2019; Boström-Einarsson et al., 2020). As a result, ARs can rarely withstand dynamic and turbulent oceanic conditions. The structures can become dislodged and damage nearby reef communities during hurricanes and storm surges. Despite their limitations, ARs are valuable tools for conducting controlled scientific experiments on reefs and are cost-effective relative to coral transplantation projects or the formation of marine reserves (Abelson, 2006; Higgins, 2019).

Sint Maarten’s marine ecosystems have been degraded by overfishing, tropical storms, runoff, and a suite of other compounding stressors over the past five decades (Mitchell, 2018). Like many ecosystems in the Caribbean, coral reef communities in Sint Maarten have been severely affected by hurricane activity. Hurricane Irma (September 2017) caused widespread damage to the island and is one of the strongest hurricanes to have hit in the Atlantic Ocean to date, with winds exceeding 185 mph (Cangialosi, Latto & Berg, 2018). Large storm surge caused physical damage to the reefs and intense rainfall increased freshwater and nutrient input (Mitchell, 2018). Sint Maarten coral communities also suffer from continued deterioration due to eutrophication, increased sedimentation, and pollution from boating traffic (Mitchell, 2018).

**Figure 1:**
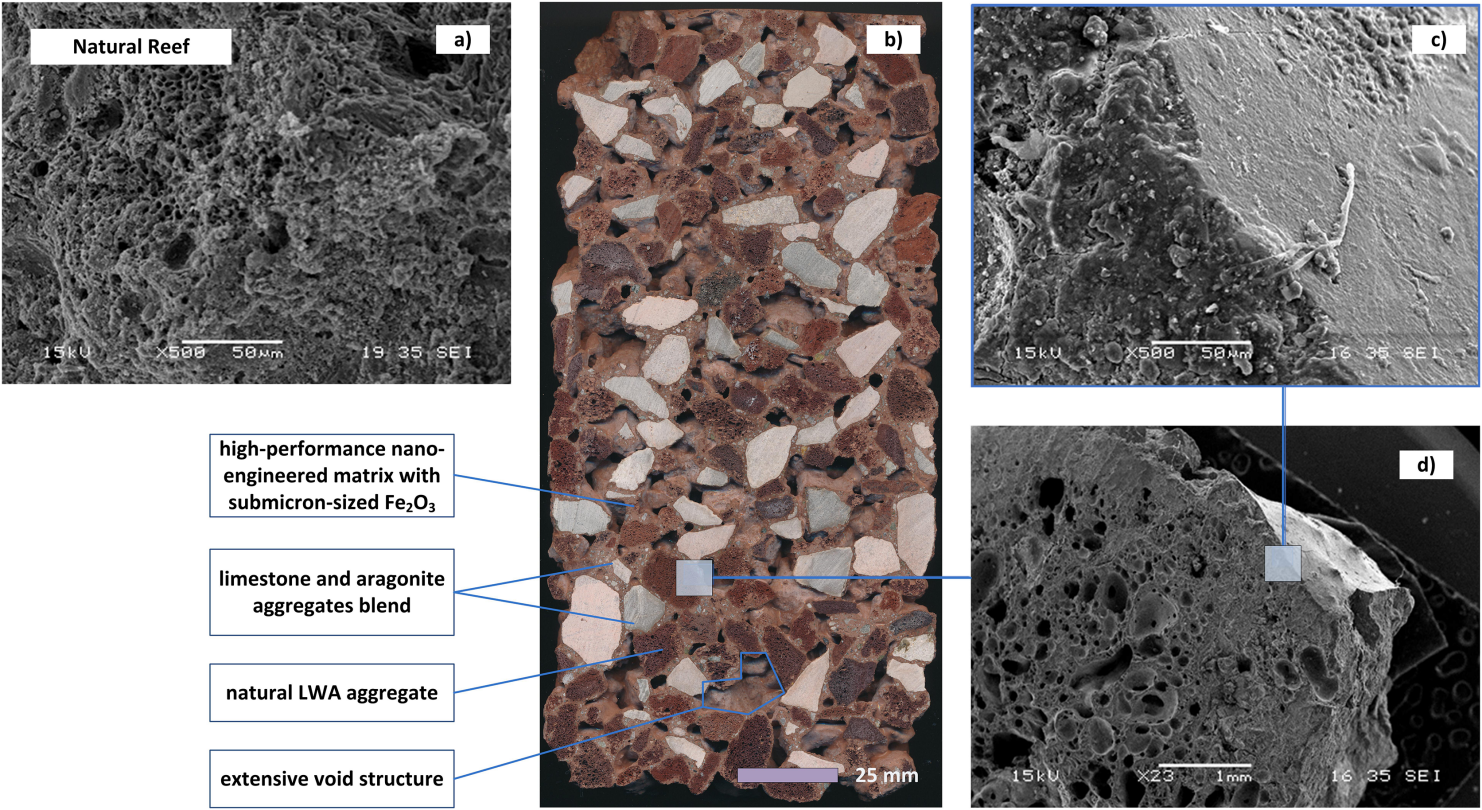
The design and structure of Oceanite substrate as compared to natural reef material. (A) The structure of natural reef material (Scanning Electron Microscope image, top left) as compared to (B) the structure of Oceanite substrate, which is designed using (C) a very dense cementitious glue bonding (D) porous LWA inclusions and other aggregates providing interconnected void structure.

Reported biomimicking mineral habitats support diverse biological communities and protect coastlines by employing nano-engineered cementitious composites called “Oceanite” tuned to marine-specific reef growth applications. The Oceanite family of AR substrates is composed of inorganic binder matrices that further include high-grade pozzolanic aggregates, combined with diverse reef-building mineral components, Fig. 1 as discussed by the authors (Yeğinobali et al., 1998; Sobolev, 2003, 2004, 2005; Sobolev & Yeğinobali, 2005; Flores-Vivian et al., 2017). Oceanite substrates were designed to enable precision control of surface pH, chemistry, and texture to attract target marine species and enhance the growth of calcareous organisms. Oceanite can be chemically and structurally customized for particular AR objectives. For example, the substrates can have an extensive interior pore network for enhancing colonizable surface area and the substrate microtopography and mineralogy can be designed to facilitate species-specific coral recruitment and survival.

Three AR structures made from Oceanite were deployed in November 2018 in Philipsburg, Sint Maarten to test the performance and biological applications of the mineral substrates in an ocean environment for the first time. The deployment goals for the ARs included: creating complex fish habitat, food, and shelter, providing shaded areas for cryptic invertebrate communities, increasing small-scale site biodiversity, and adhering to strict budgetary and logistical requirements. The pilot study had three main objectives: (1) to quantify and compare the sessile benthic communities growing on the three structures, (2) to qualify fish behaviour on ARs and compare approximate fish biomass to adjacent reef communities, and (3) to create a preliminary species identification catalogue for organisms growing on Caribbean Oceanite substrates.

## Materials and Methods

### Substrate material

The design for the Oceanite mineral matrices followed the established guidelines discussed by the authors (Yeğinobali et al., 1998; Sobolev, 2003, 2004, 2005; Sobolev & Yeğinobali, 2005; Flores-Vivian et al., 2017). The composition of Oceanite combines pervious cast stone concepts (Wanielista & Chopra, 2007) with high-performance natural Light-Weight Aggregate (LWA) concrete (Yeğinobali et al., 1998) and ultra-high-performance nano-engineered concrete matrices (Sobolev, 2004; Flores-Vivian et al., 2017; Muzenski, Flores-Vivian & Sobolev, 2019). The engineered substrates (Fig. 1, center) can be tuned to promote reef restoration by controlling near-surface pH, leachate emissions, surface texture, porosity, void structure, and can also possess hydrophobic, photocatalytic, and antimicrobial properties (Hosseini et al., 2013; Faraldos et al., 2016). The control of near-surface pH is achieved by the incorporation of submicron and nano-sized particles of pozzolanic (amorphous) minerals. Achieving a very effective mixture of the composite binder includes a very low water-to-cementitious ratio, composed of a range of particles from 5 nm to 95 microns. The resulting cementitious mixture was characterized by a dense matrix (Fig. 1, right) with very high strength, in the range of 125–145 MPa and exceptional durability under extreme exposures (Muzenski, Flores-Vivian & Sobolev, 2015; Muzenski et al., 2020; Sobolev et al., 2016). These very high-strength composites were extensively tested for low-temperature applications, including freezing to −50 °C and subsequent thawing and de-icing salt scaling (Muzenski, Flores-Vivian & Sobolev, 2015). The excellent performance of similar compositions was also demonstrated in tropical environments (Castro-Borges, Balancán-Zapata & Zozaya-Ortiz, 2017). For a selected group of ARs (50% of manufactured volume), the resulting cementitious matrix was further doped with up to 5% of submicron-sized minerals, formulated to attract marine species.

**Figure 2:**
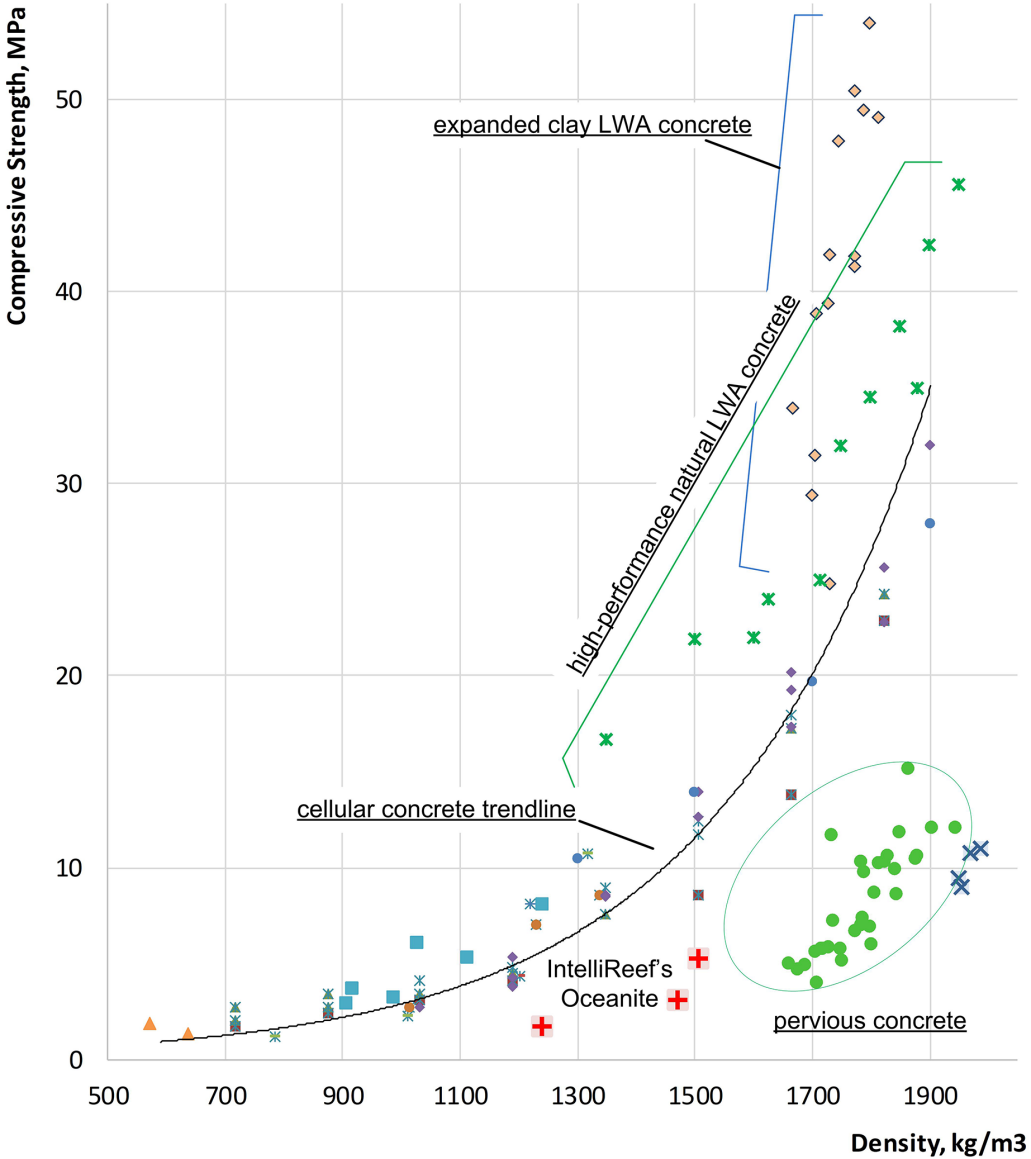
A comparison of the strength-density relationship for a range of light-weight concrete materials as compared to Oceanite. The strength-density relationship for a range of light-weight concrete materials as compared to Oceanite: expanded clay LWA concrete (Kockal & Ozturan, 2004); high-performance natural LWA concrete (Yeğinobali et al., 1998); pervious concrete (Wanielista & Chopra, 2007).

Many underwater applications, such as ARs, would not require very high strength; but rather fine-tuning of mix designs for biocompatibility and marine community retention. This is achieved by designing the material with an extensive void structure, enabling the substrate to act as a bio-filter and reduce the structure’s loading from waves and currents. Substrate porosity was prioritized to achieve a surface area needed for biodiversity enhancement and biofilter applications. Desired filtration results for Oceanite were achieved when coarse mineral aggregates with a maximum size of 25 mm were coated with a relatively small volume of cement paste. The resulting composite had a dry density of 1.5 kg/liter (*vs*. conventional concrete ranging from 2.3 to 2.4 kg/liter), which enabled internal void percolation and excellent permittivity, as required for water filtration. Here, the microtopography of the volcanic rock was hypothesized to be favorable for the settlement of coral larvae and other reef-building species. It can be observed that the strength of the resulting Oceanite substrate material (IntelliReefs’ second generation of Oceanite) was at the target level of 5 ± 0.15 MPa, as demonstrated by Fig. 2. This minimum strength level was selected to meet the structural requirements for masonry units and still maximize the filtration ability of the AR. Due to incorporated open-void structure, the strength of the substrate was lower than commonly observed for cellular concrete of the same density (such as IntelliReefs’ first generation of Oceanite products tested in Hawaii 2017), but due to the use of a very high strength cementitious matrix, the minimum design strength was achieved at lower density *vs*. observed in pervious concrete (1.5 *vs*. 1.7 kg/liter, respectively).

The freshly mixed underwater Oceanite masonry units were pre-cured in molds for 15 h, demolded, and cured for 30 days in the greenhouse under temperature conditions of 30 ± 7 C and relative humidity of 80 ± 7%. The representative samples were collected and tested for density and compressive strength (Fig. 2). The manufactured units were inspected and placed on pallets for follow-up shipping. Since the installation happened only 4 months after the final curing, this additional time was helpful for the substrate material to achieve a complete hardening under internal curing conditions. Based on the described procedure, the Oceanite-marine composite reefs can be designed and tuned up for site-specific conditions with regard to biological and physical requirements.

### Deployment sites and background communities

**Figure 3:**
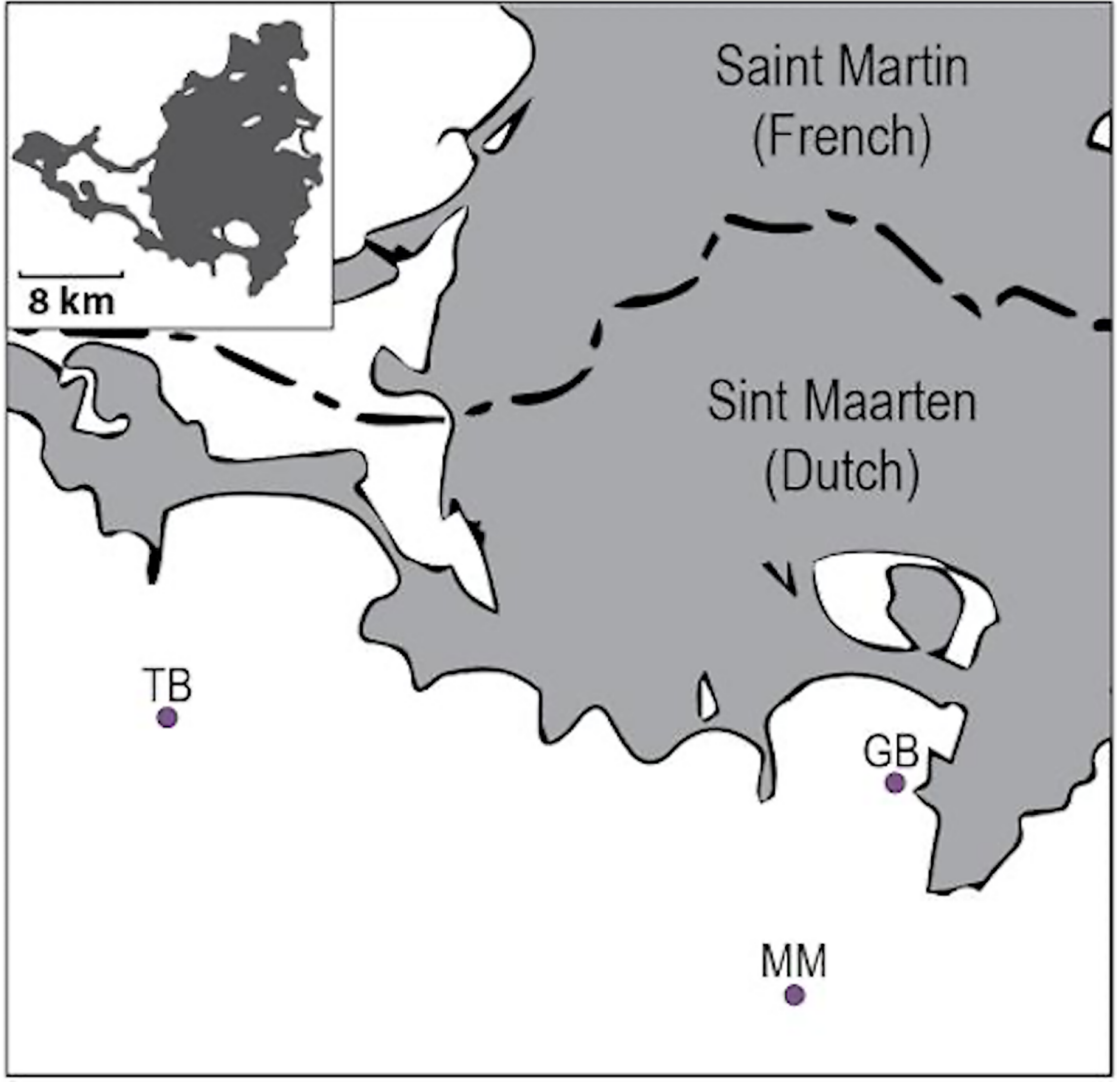
Map of Sint Maarten (Dutch section) showing the location of study sites. Map of Sint Maarten (Dutch section) showing the location of study sites: Mike’s Maze (MM), The Bridge (TB), and Great Bay (GB). Inset shows the entire island with scale.

**Figure 4:**
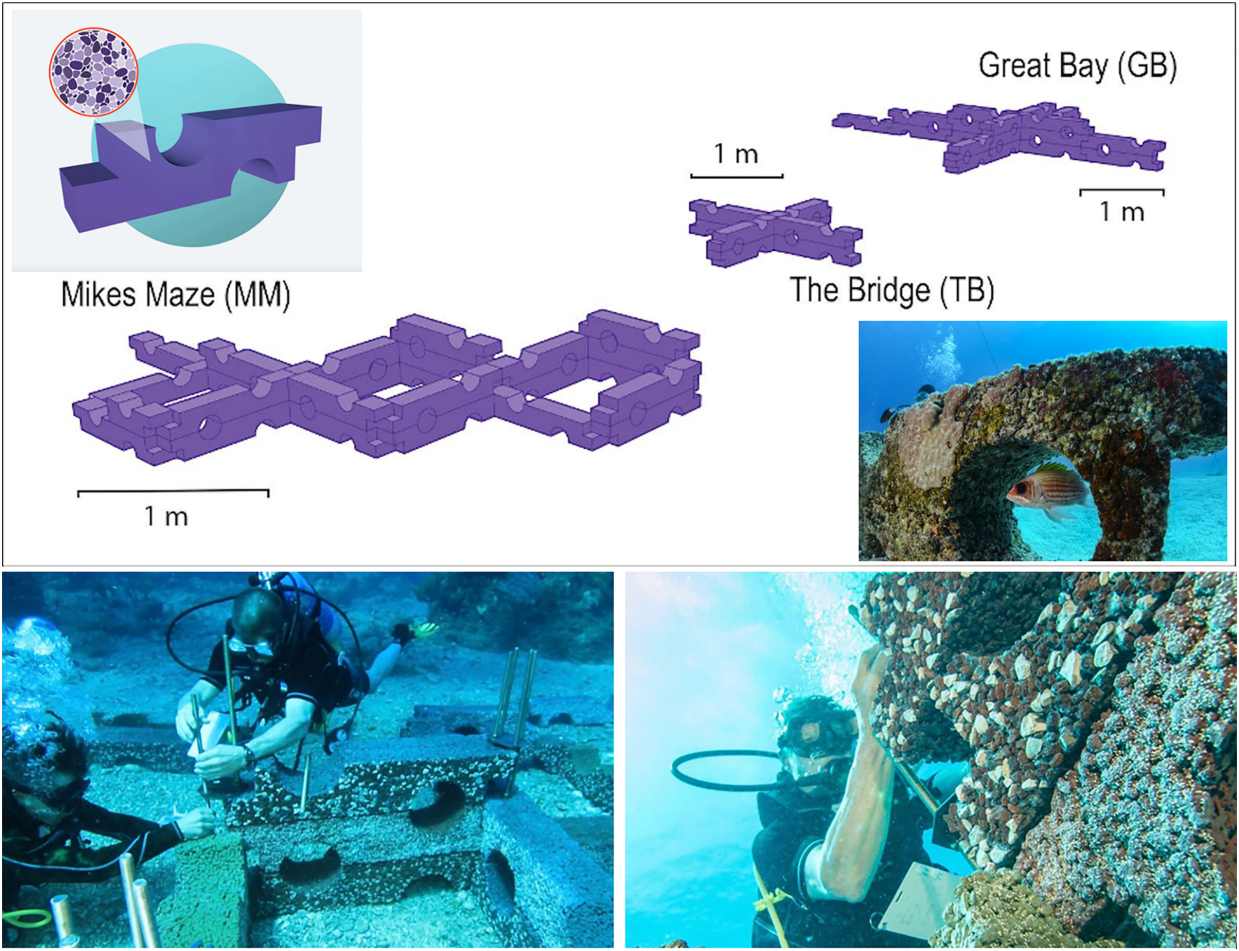
Modular Oceanite artificial reef structures deployed off the coast of Philipsburg, Sint Maarten. Oceanite artificial reefs deployed in a pilot study at Mike’s Maze, (MM) The Bridge, (TB), and Great Bay (GB) in Philipsburg, Sint Maarten.

We measured community colonization of algal and invertebrate taxonomic groups on three seafloor ARs after 14 months (October 2018-January 2020) in Philipsburg, Sint Maarten, as a pilot study to examine the performance of Oceanite (Fig. 3). Concurrent fish observations at all three study sites were conducted in January 2020. The study sites were chosen for the initial deployment locations by the team and the Sint Maarten Nature Foundation based on logistical deployment constraints and the level of exposure to environmental stressors (*i.e*., boat traffic and runoff). One AR (Mike’s Maze, MM, 25.4 m^2^; Fig. 4) was deployed at a depth of 13 m in a coral reef marine protected area (MPA) that limits boat traffic (N17.59.488, E063.03.396). The second AR (The Bridge, TB, 8.1 m^2^; Fig. 4) was deployed at 15 m in a mixed coral reef/seagrass bed through-way outside of the MPA (N18.01.273, E063.06.802). The third AR (Great Bay, GB, 14.2 m^2^; Fig. 4) was deployed at 4 m on a heavily disturbed seagrass bed in Great Bay, Philipsburg (N18.01.054, E063.02.784). The water quality in Great Bay is poor, with runoff from a local landfill directed into the Bay (Mitchell, 2018). Cruise ships also pass through the bay and are moored ~500 m from the AR site, further reducing water quality and increasing sedimentation rates (Mitchell, 2018).

The natural reef adjacent to Mike’s Maze was sampled with a video transect conducted by divers swimming parallel to and at a constant distance from the seafloor (Intova Sport HD 2). A 15 × 0.35 m video belt-transect was conducted by divers swimming along a 13 m depth contour. Divers maintained the camera at a fixed distance (1.5 m) above the seafloor and a tape measure running the length of the transect was used to provide scale. The Sint Maarten Nature Foundation conducts comparable annual site surveys at Mike’s Maze. In particular, benthic and fish community surveys conducted before and after Hurricane Irma (September 2017) provide important historical context and background metrics for our pilot study.

Videos were imported into Adobe Premiere Pro 2020 where contiguous, non-overlapping frame captures were extracted and selected for analysis using a random number generator to quantify the percent cover of algae, stony corals, soft corals, and other sessile benthic invertebrates. The uncolonized frame area consisted of bare (*i.e*., uncolonized) substratum and sand (at the natural reef). Frame captures on the natural reef (NR: *n* = 10) encompassed 28.8 m^2^ of the seafloor. Still images of the horizontal (TB-H: *n* = 2; GB-H: *n* = 2; MM-H: *n* = 3) and vertical substrates (TB-V, GB-V: *n* = 4; MM-V: *n* = 6) encompassed ~25% of the Euclidean surface area of each substrate orientation for each AR, respectively. All images were analyzed using ImageJ (1.52q). The percent cover of taxonomic groups (see “Sampling and Analysis of Artificial Reef Communities” Section) was calculated by overlaying 100 uniformly spaced points across the image.

### Sampling and analysis of artificial reef communities

To quantify the biotic assemblage on the ARs at our study sites, divers photographically surveyed each structure using an underwater camera (Nikon D7000) in January 2020. All horizontal (top) and vertical (sides) surfaces of each AR were sampled with flash photography taken by divers swimming ~0.5 m from the substrates. The deployed AR modules were of a known size and provided scale for the photographs.

For both experiments, still photographs of horizontal and vertical surfaces (image size: 4,000 × 3,000 pixels, 4.3 MB) were analyzed using ImageJ (1.52q) by overlaying 100 uniformly spaced points across the AR substrate to measure the percent planar cover of each taxonomic group of colonists. Points were excluded where the substrate was obscured by a motile invertebrate. Microbial or algal taxonomic groups included biofilms (bacteria, microalgae), epilithic algal matrix (conglomerate of filamentous turf-forming algae, sediment, and detritus), macroalgae (non-coralline fleshy or foliose brown and green algae, *e.g*., *Dictyota* spp., *Lobophora* spp.), and encrusting crustose coralline algae (CCA). Invertebrate groups included: ascidians, anemones, bivalves, bryozoans, corals, hydrozoans, and polychaetes.

Species were identified to the lowest taxonomic level from high-resolution photographs and video records. A photographic catalogue from each site was kept for all macroalgal and invertebrate taxa. Samples of individuals that could not be identified to genus or species based on available taxonomic keys or local expertise were sent to experts for assistance.

### Statistical analysis

Given the difference in surrounding ecosystems at each deployment site and varying AR depths, primarily observational data was gathered by this pilot study. However, we did statistically compare total percent planar cover (%) between MM, TB, and GB, respectively on horizontal and vertical substrates separately using unpaired t-tests (α = 0.05). The total percent planar cover (%) at the natural reef community at MM was also compared to horizontal substrates at MM Oceanite AR structure using unpaired t-tests (α = 0.05). The mean frequency of occurrence for all fish species was compared between MM and TB using unpaired t-tests. Bite frequency data was unreplicated, and no statistical tests were used to compare bite rates among sites.

### Fish community analysis and behaviour

Reef fish were opportunistically monitored between 28 and 30 January 2020 using static videography at MM, TB, and GB and the results are reported in Tables 1 and 2. At each site, cameras (GoPro Hero 3, GoPro Hero 7) in continuous video mode were fixed ~0.75 m above the seafloor on underwater monopods for 10–15 min on ARs (Table 2). Video records were analyzed to identify fish species (presence/absence), frequency of occurrence, behaviour (grazing, territorial defense, sheltering), and feeding frequency for individuals >5 cm (bite rates per video record, all species combined). Abundance was measured by frequency of occurrence from individual video frames (*n* = 10) extracted at 60-s intervals from each site in Adobe Premiere Pro 2020. The first and last minutes of each video were removed as a buffer for potential effects of diver presence (divers returned to the boat or left the site during sampling). Fish were observed to resume normal foraging behaviour on the structures within ~1 min of divers’ departure once recording had begun, particularly herbivorous fish with a greater potential for disturbance (*e.g*., surgeonfish).

**Table 1:** Species identification table for three Oceanite artificial reefs near Philipsburg, Sint Maarten. Data are the lowest taxonomic level (Cl, Class; Or, Order; Fa, Family) identified* for algae and invertebrates on horizontal (H) and vertical (V) Oceanite artificial reef (AR) substrates after 14-mo of deployment at two seagrass beds (TB, GB) and a coral reef inside a marine protected area (MM) in Philipsburg, Sint Maarten.

Phylum/Class	Order/Family	Genus/Species	Site-Orientation
Porifera			
Cl Calcarea			
Subcl Calcaronea	Or Leucosolenida		
	Fa Grantiidae	*Leucandra aspera*	GB-V
Cl Demospongiae			
Subcl Heteroscleromorpha	Or Poecilosclerida		
	Fa Desmacididae	*Desmapsamma* sp.	TB-V
	Or Haplosclerida		
	Fa Petrosiidae	*Neopetrosia carbonaria*	TB-V
Cnidaria			
Cl Anthozoa	Or Scleractinia		
	Fa Poritidae	*Porites astreoides*	MM-H, MM-V, TB-V
Cl Hydrozoa	Or Anthoathecata		
	Fa Penariidae	*Pennaria disticha*	GB-V
	Or Anthoathecata		
	Fa Milleporidae	*Millepora alcicornis*	MM-H
Subcl Hydroidolina	Subcl Leptothecata		
	Superfa Plumularioidea		
	Fa Aglaopheniidae	*Gymnangium allmani*	TB-V
Bryozoa			
Cl Gymnolaemata	Or Cheilostomatida		
	Subor Flustrina		
	Superfa Celleporoidea		
	Fa Colatooeciidae	*Trematooecia aviculifera*	TB-V, MM-V
Annelida			
Cl Polychaeta			
Subcl Sedentaria			
Infracl Canalipalpata	Or Sabellida		
	Fa Sabellidae		
	Subfa Sabellinae	*Bispira variegata*	GB-V
Chlorophyta			
Subph Chlorophytina			
Cl Ulvophyceae	Or Bryopsidales		
	Fa Caulerpaceae	*Caulerpa* sp.	GB-H, GB-V
Ochrophyta			
Cl Phaeophyceae	Or Dictyotales		
	Fa Dictyotaceae	*Dictyota* sp.	GB-H, GB-V
		*Lobophora* sp.	GB-V, MM-V
Chordata			
Subcl Tunicata			
Cl Ascidiacea	Or Enterogona		
	Subor Phlebobranchia		
	Fa Perophoridae	*Ecteinascidia* sp.	GB-V
	Or Stolidobranchia		
	Fa Styelidae	*Botrylloides* sp.	GB-V
		*Polycarpa spongiabilis*	MM-V
	Or Aplousobranchia		
	Fa Holozoidae	*Distaplia* sp.	GB-V
	Fa Didemnidae	*Trididemnum soildum*	GB-V

**Note:**

* Identification References and Experts: Algaebase, WoRMS, Reefbase, Taxonomic Key, Melanie Meijer zu Schlochtern (Nature Foundation, Sint Maarten); *Poriferans*: WoRMS, Taxonomic Key.

**Table 2:** The abundance, species present, and behaviour of fish species on three Oceanite artificial reefs near Pilipsburg, Sint Maarten.

Fish species	Mean frequency of occurrence	Bite Rates (bites min^−1^)	Fish behaviour
*Mike’s Maze (MM)*			
*Acanthurus* spp.	2.00	2.11	Grazing
*Abudefduf saxatilis*	0.80	0.77	Grazing, sheltering
*Stegastes partitus*	0.40	0.64	Grazing, sheltering
*Mycteroperca* sp.	0.00	0.13	Grazing
*Thalassoma bifasciatum*	0.00	0.06	Grazing
*Scarus* spp.	0.00	0.06	Grazing
*Halichoeres* sp.	0.00	0.06	Grazing
*Aluterus* spp.	0.00	0.06	Grazing
*Pseudupeneus maculatus*	0.10	0.00	Present near structure
*Mulloidichthys martinicus*	0.10	0.00	Present near structure
*Chaetodon striatus*	0.00	0.00	Present near structure
*Chaetodon capistratus*	0.00	0.00	Present near structure
*Haemulon flavolineatum*	0.00	0.00	Present near structure
Total	3.40 ± 0.43	3.89	
*The Bridge (TB)*			
*Thalassoma bifasciatum*	35.50	1.15	Sheltering, grazing
*Acanthurus coeruleus*	1.00	0.46	Grazing
*Stegastes partitus*	3.50	0.46	Sheltering, grazing
*Acanthurus* spp.	0.20	0.35	Grazing
*Holocentrus adscensionis*	0.90	0.12	Sheltering
*Pseudupeneus maculatus*	2.30	0.12	Foraging, grazing
*Caranx latus*	0.00	0.00	Present near structure
*Mulloidichthys martinicus*	2.00	0.00	Foraging, sheltering
*Sphyraena barracuda*	0.10	0.00	Present near structure
Total	45.5 ± 3.28	2.66	

Note:

Data are species present, mean frequency of occurrence (±SE), feeding frequency (bites min^−1^), and behaviour observed for all fish species on Oceanite artificial reefs (ARs) after 14-mo of deployment at two seagrass beds (TB, GB) and a coral reef (MM) inside a marine protected area (MPA) in Philipsburg, Sint Maarten based on video records from January 28–30 2020. Data are mean ± SE for video records (*n* =10) in which fish frequency of occurrence was calculated.

## Results

### Cover of the colonizing communities at the artificial and natural reefs

**Figure 5:**
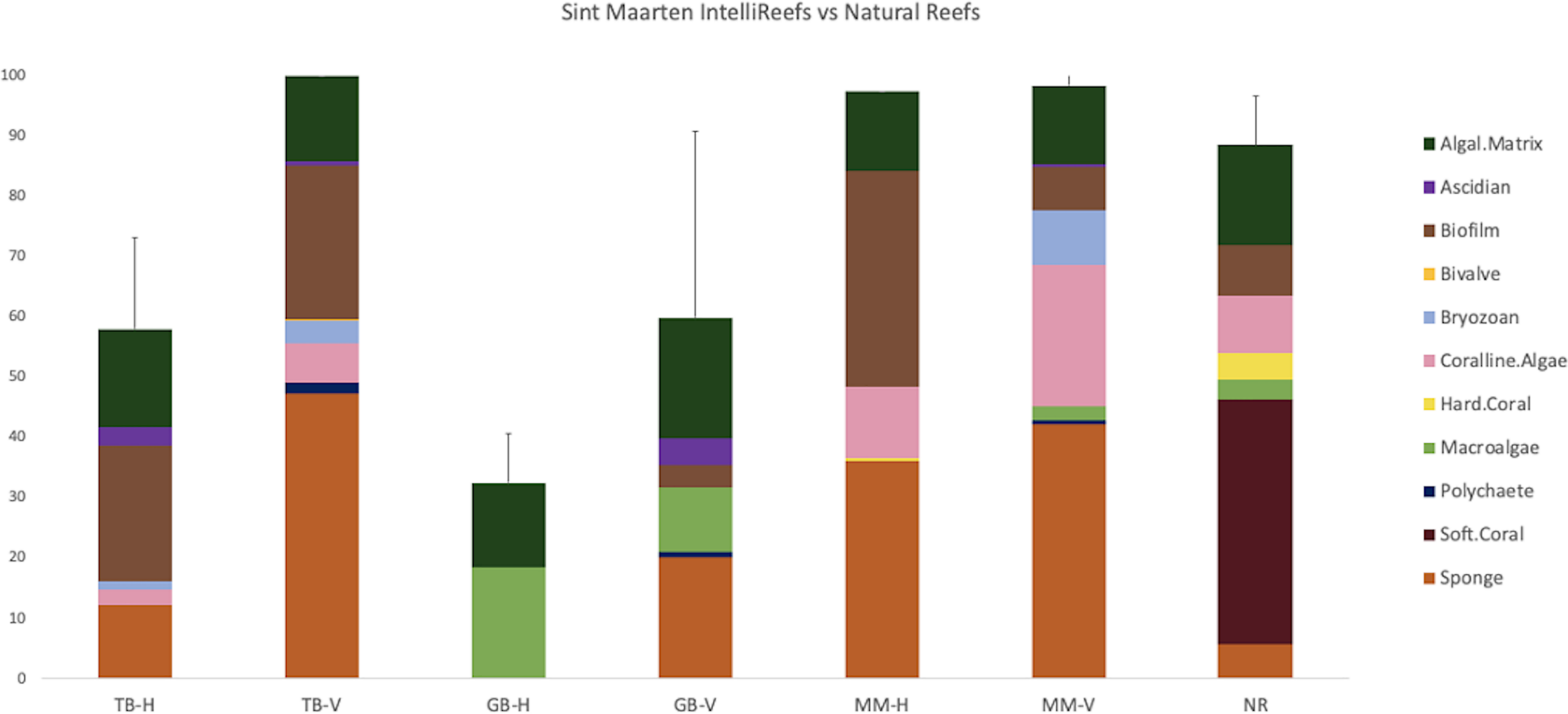
Community composition on artificial reefs and the natural reef adjacent to Mike’s Maze (MM). Mean (+SD) planar cover (%) and composition of sessile benthic organisms on horizontal (top) substrates (MM-H, TB-H, GB-H: *n* = 2 frames) and vertical (side) substrates (MM-V, TB-V, GB-V: *n* = 4 frames) on Oceanite artificial reefs and upper substrates of the natural reef (MM-N: *n* = 10 frames), in January 2020.

After 14 months of operation, all three Oceanite ARs deployed off the coast of Philipsburg, Sint Maarten were heavily colonized by invertebrate and algal species (Fig. 5). The total cover of the benthic community on the AR horizontal substrates at all sites ranged from 32.5–97.4%. Total horizontal cover on MM was significantly higher than both TB and GB (TB: t_4_ = 4.522, *P* = 0.011; GB: t_4_ = 13.815, *P* = 0.0002). Cover on horizontal substrates at TB and GB was not significantly different (t_4_ = 2.592, *P* = 0.061). The natural coral reef adjacent to MM had a total cover of 89.8% and was not significantly different from horizontal substrates at MM (t_4_ = 1.641, *P* = 0.176). The cover of benthic invertebrates on horizontal substrates, including sponges, ascidians, bryozoans, and corals, was low at MM (36.6%) and TB (16.4%) and negligible at GB. Macroalgae were observed on horizontal substrates only at GB (18.6%).

On vertical substrates, total cover ranged from 59.9–100.0%. Vertical substrates at TB had significantly more total cover than their corresponding horizontal substrates (TB, t_4_ = 6.425, *P* = 0.003), while there was no difference between substrate types at MM and GB (MM, t_7_ = 0.645, *P* = 0.540; GB, t_5_ = 1.466, *P* = 0.203). Total planar cover was not significantly different between MM-V and TB-V (t_8_ = 1.170, *P* = 0.276), but both TB-V and MM-V were significantly different from GB-V (TB, t_6_ = 2.587, *P* = 0.041; MM, t_8_ = 3.125, *P* = 0.014). Cover of all benthic invertebrates on vertical substrates was highest at TB (54.0%) and MM (52.3%) and largely dominated by sponge species (MM, 42.2%; TB, 47.3%). Macroalgae was rare on vertical substrates at GB (10.5%) and TB (2.3%) and negligible at MM.

The structures supported a diverse array of invertebrate colonists on vertical substrates, with dominant sponge species identified at MM, GB, and TB from the families *Petrosiidae*, *Desmacididae*, and *Grantiidae*. Solitary and colonial ascidians present on AR topsides were from *Perophoridae*, *Styelidae*, *Holozoidae*, and *Didemnidae* families. Identified bryozoans present on vertical and cryptic substrates (under ledges) were *Trematooecia aviculifera*. Macroalgal species were only observed at GB; species observed were *Caulerpa* sp., *Dictyota* sp., and *Lobophora* sp.; coral recruits were observed by divers on both horizontal and vertical substrates at MM and TB were *Porites astreoides* (Table 1).

### Fish behaviour

Communities at all Oceanite ARs were video-monitored for frequency of occurrence, species composition, feeding frequency, and behaviour from 28–30 January 2020. The difference in mean total frequency of occurrence was found to be extremely significantly different between TB and MM (t_18_ = 12.727, *P* < 0.001) (Table 2). No fish were observed in video footage from GB due to poor water quality, but diverse assemblages of fish were observed at MM and TB.

A greater diversity of fish species was observed on or near (<2 m away) the AR structure at MM than TB. The most common fish species observed biting at MM were ocean surgeonfish and doctorfish (*Acanthurus* spp.), sergeant majors (*Abudefduf saxatilis*), and bicolour damselfish (*Stegastes partitus*) (Table 2). Sergeant majors (*Abudefduf saxatilis*) and bicolor damselfish (*Stegastes partitus*) were the only species observed sheltering on AR at MM (Table 2). Spotted and yellow goatfish (*Pseudupeneus maculatus* and *Mulloidichthys martinicus*) frequently foraged on AR at TB; bicolour damselfish (*Stegastes partitus*) were frequently observed grazing at TB, along with bluehead wrasse (*Thalassoma bifasciatum*) and blue tang (*Acanthurus coeruleus*) (Table 2). At TB, sheltering fish species include bluehead wrasse (*Thalassoma bifasciatum*), bicolor damselfish (*Stegastes partitus*), squirrelfish (*Holocentrus adscensionis*), and yellow goatfish (*Mulloidichthys martinicus*) (Table 2). Divers observed two gobies (*Coryphopterus* sp.) sheltering in the AR at GB, but they were not visible in fish monitoring videos.

## Discussion

### Community composition on deployed ARs and natural reef

Evaluating the efficacy of advanced material substrates for enhancing biological and economic objectives on coral reefs is essential for elucidating their large-scale applications in coral reef restoration. The initial results from our observational Oceanite AR pilot study in Philipsburg, Sint Maarten indicate a potential for using proposed AR substrates to increase local biodiversity and create optimal conditions for rapid species growth by engineering the physical and chemical design of the structures. After 14 months underwater, the developed ARs were covered in a vibrant community of benthic plants and animals. The study proved that the horizontal substrates of ARs were dominated by algal and sponge species, and vertical substrates supported a diverse assemblage of suspension- or filter-feeding invertebrates (*e.g*., sponges, bryozoans, ascidians, *etc*.). For all ARs, the total percent cover of benthic invertebrates and calcifying organisms was higher on vertically oriented substrates than on horizontally oriented substrates (Fig. 5). It was observed that the community composition differed between the AR in the MPA and the adjacent protected natural reef dominated by soft corals. Crustose coralline algae (CCA) cover was highest on the AR in the MPA, with both surface orientations having more CCA than the adjacent natural coral reef which had only 9.5% CCA coverage. Here, CCA is a group of calcifying algal species that facilitate coral settlement through the formation of calcium carbonate and microbial communities, supporting healthy coral reef development and facilitating coral metamorphosis (Barott & Rohwer, 2012; Dean et al., 2015). We found that Oceanite CCA coverage was higher than comparable methods used in nearby Belize (Arnold & Steneck, 2011). For example, after 14 months of deployment, Arnold & Steneck (2011) found the terra-cotta settlement plates (the globally preferred coral recruitment material) had accumulated only 13% CCA coverage. Oceanite mixtures are specifically designed to attract calcifying organisms (*e.g*., corals, CCA, bivalves, polychaetes, *etc*.). Finding high levels of CCA on the structures suggests that the AR structures are performing as expected and have the potential to be scaled up in future projects to facilitate the settlement of reef-building species on a regional scale.

Corals reproduce by brooding and releasing larvae or broadcasting gametes into the water column (Szmant, 1986). Coral larvae actively choose where to settle on the hard substrate based on light, chemical, physical, temperature, density, and auditory cues (Mundy & Babcock, 1998; Babcock & Smith, 2002; Birrell, McCook & Willis, 2005; Vermeij & Sandin, 2008; Vermeij et al., 2010). After the larvae choose a suitable substrate, they still must endure a high probability of mortality in their early life stages due to grazing by predators and overgrowth through competition for available space (Sammarco, 1980; McCook, Jompa & Diaz-Pulido, 2001; Christiansen et al., 2009). Divers observed macroscopic coral recruits (*Porites astreoides*) at both ARs deployed in the MPA (MM) and the through-way (TB). This supports the project hypothesis that the developed AR structures can facilitate wild coral settlement in a relatively short amount of time. Based on the number of polyps on recruits, the corals settled during their last reproductive season (May–September 2019), less than 1 year after AR deployment. Here, complex microtopography and porosity of Oceanite may help address early life stage mortality by providing shelter through cryptic spaces and increased surface area. Our next phase of research will include a standardized experiment to measure coral recruitment and survival on Oceanite and other state-of-the-art artificial reef materials (*i.e*., concrete, steel, terra-cotta, coral skeleton) using replicated settlement tiles.

The reef site at Great Bay is exposed to the most boat traffic of all three sites and experiences a high amount of sedimentation and abrasion due to its shallow depth, turbulent waters, and sediment composition. As a result, Great Bay had the lowest total cover of benthic invertebrates on both horizontal and vertical substrates at all three sites (Fig. 5). Though there was a decrease in benthic invertebrate coverage, the substrates at Great Bay had the highest algal cover at all sites, suggesting that algae species in this area are more tolerant of the environmental conditions than benthic invertebrates (Fabricius & Wolanski, 2000; Orme, 2012). Surrounding benthic communities and site background data also affect invertebrate colonization patterns (Barott & Rohwer, 2012), underscoring the importance of compiling environmental data and concurrently monitoring adjacent communities in AR studies. These results are key for considering site-specific design considerations for turbulence in compromised ecosystems (*e.g*., higher vertical relief and walls buffering sheltered internal communities) in future AR deployments.

A recent study in Sydney, Australia, has demonstrated that purpose-built artificial reefs, in collaboration with other conservation initiatives, can increase fish populations and biomass (Folpp et al., 2020). This reported study also examined the fish abundance and feeding behaviour of the ARs. Grazing rates are highly dependent on the abundance and composition of fish communities (Mumby & Hastings, 2008). At Mike’s Maze, located in the MPA, fish abundance and grazing rates were lower than at The Bridge (Table 2). Herbivorous and omnivorous fish fed directly off AR structures at both Mike’s Maze and The Bridge, indicating that the ARs are food sources for resident fish (Table 2). Regional grazing rate comparisons are notoriously confounded by local differences in fishing pressure (Hay, 1984) and have not been documented on the coral reef in the MPA. Therefore, comparisons can be drawn between the natural reef and the AR at this time. The research team is in the process of coordinating further data collection in partnership with Sint Maarten’s Nature Foundation, which is the local MPA management organization. We found that the fish community at Mike’s Maze was more diverse than at The Bridge, but as the surrounding benthic communities are different at these sites (*i.e*., coral reef *vs*. mixed coral reef and seagrass bed), we cannot disentangle differences in fish community composition between the two sites. While water quality was too poor to examine the fish abundance and behaviour on the AR at Great Bay, divers observed two gobies sheltering in the structure.

Our study found that the AR in the unprotected through-way (The Bridge) had an order of magnitude more fish than MM in the MPA. Increased fish abundance at The Bridge may be due to the shipwrecks (>3 vessels) located adjacent to the AR. The additional three-dimensional complexity provided by these structures has the potential to attract and recruit fish from the water column and the surrounding coral reefs and seagrass bed (Pickering & Whitmarsh, 1997). Both pelagic and demersal fish species have been found to seek shelter in high-relief ARs relatively soon after deployment, indicating that ARs can be effective at mitigating the effects of overfishing and rehabilitating depleted fish stocks (Rilov & Benayahu, 2002). However, this is contingent on the simultaneous reduction of fishing pressure in the area (Wilson, Leung & Kennish, 2002). For this reason, large ARs with significant vertical relief deployed in MPAs that prohibit or severely limit fishing have the potential to mitigate the effects of overfishing. These findings at The Bridge also suggest that the ARs in areas that are deployed in a network of high-relief structures (artificial or natural) may increase fish colonization and abundance around ARs.

The reported pilot study was beholden to several site and resource limitations. Sites were selected largely due to ease of deployment and to assess Oceanite mineral matrices under a variety of environmental conditions. As such, funding and deployment personnel were limited, and we were not able to replicate ARs for each site treatment level (*i.e*., coral reef, mixed habitat, seagrass bed). As such, analyses were limited to observational comparisons and individual statistical tests per treatment (*i.e*., between the AR on a coral reef and the adjacent natural reef) rather than a comprehensive statistical comparison across all sites. Due to variations in background communities at each AR site, benthic community differences may be confounded by levels of exposure to fish and invertebrate consumers. Future research on the proposed AR concept will involve replicated deployment of structures by site treatments and concurrent video monitoring of motile consumers. Given the adjacent background community, understanding the relationship between colonization patterns on AR structures will inform future design and monitoring protocols.

### The potential advantages of artificial reefs based on advanced materials

Advanced nano-engineered AR material to target biological and economic conservation goals have the potential to enhance both direct and indirect socio-economic benefits with positive externalities ranging from private sector tourism, commercial fishing, and coastal development to public ecosystem services such as coastal protection, artisanal fishing, and cultural practices (Seaman, 2002; Thanner, McIntosh & Blair, 2006; Jan et al., 2003; Ali et al., 2013). Remediating or enhancing biological variables (*i.e*., increasing fish, biodiversity, and coral cover) using advanced material ARs would create substantial economic benefits. A study reported by the United Nations Environment Programme focusing on the Mesoamerican Reef and the Coral Triangle predicts that a shift towards healthy coral reefs in these regions could provide economic benefits valued at $34.6 billion and $36.7 billion respectively, across tourism, coastal development, and commercial fisheries sectors by 2030 (International Coral Reef Initiative, 2018).

Thriving reefs create direct benefits for commercial and artisanal fisheries, including reliable catch rates due to stable fish populations (Baine, 2001; Jan et al., 2003; Ali et al., 2013). Indirect benefits include the creation of jobs in fish processing, marketing, boat building, and net making (Burke et al., 2004). If reefs continue to deteriorate, the fishing industry will be crippled, causing the loss of thousands of jobs in the Carribean alone (Burke et al., 2004). The Oceanite ARs in this study yielded nearly four bites per minute from fish species, and ARs have been known to attract both foreign fish and recruits from nearby deteriorated reefs (Rilov & Benayahu, 2002; Higgins et al., 2019). This indicates the potential for rehabilitating depleted fish stocks. An increase in fish feeding on the structure can surge local fish populations, increasing commercial and artisanal fisheries yields and countering the effects of overfishing. Furthermore, increasing fish stocks through ARs deployed in an MPA can create a significant spillover effect into surrounding, unprotected waters for fisheries (Folpp et al., 2020; Glarou, Zrust & Svendsen, 2020). ARs designed physically and chemically to enhance local fish production can assist in safeguarding local jobs through the provision of additional biodiverse benthic communities as fish feeding stations and complex, three-dimensional habitat to shelter within. Bio-enhancing ARs can also be used to attract fish to recolonize degraded natural reefs, reinforcing both job and food security. Healthy reefs not only affect the local fishing industry but create global benefits as well.

Extensive research has been conducted on the socio-cultural valuation of natural reefs (Cesar, 2000; Oles, 2007; Cinner, 2014), which can be used as a benchmark for assessing the benefits of additional habitat creation through the deployment of ARs based on advanced materials. As the deployment of ARs for coral reef restoration has increased globally (Higgins, 2019), specific interest in their socio-cultural benefits has also expanded. Chen et al. (2013) examined the valuation of 88 ARs on the island of Penghu, Taiwan. Penghu has deployed ARs since 1974 to improve water quality and support the fishing industry. While no initial socio-cultural goals were associated with the AR deployments in Penghu, researchers found that the tourism industry greatly improved, with additional economic benefits estimated at $92.6 million primarily from sport fishing and scuba diving ticket sales (Chen et al., 2013). This case study suggests that traditional ARs globally have a myriad of biological and economic benefits through additional habitat creation alone. We recommend that the implementation of advanced ARs, as described in this article, engineered specifically to address the goals of the fishing and tourism industries, could yield an even higher return on investment and circulate further benefits through the local community in both sectors. For example, ARs made from Oceanite have the added benefit of increasing local biodiversity and rapid, targeted animal settlement, building resilient reefs in the face of future environmental stressors.

The tropical tourism industry is heavily reliant on healthy coral reefs. Scuba divers make up about 10% of all Caribbean tourists but constitute 17% of all tourism revenue (Burke et al., 2004). Divers are attracted to locations with the most pristine and diverse coral reefs and are willing to pay per dive to keep coral reefs healthy (Burke et al., 2004; Hawkins et al., 2005). These funds could, in turn, be reinvested into the local community or used to maintain successful restoration projects, such as local ARs. Under a healthy reef scenario, diving tourism is expected to increase by 7% per year *vs*. 5.5% for general tourism, making the ongoing ecological integrity of global reefs a significant consideration for tourism growth (Burke et al., 2004). ARs can also contribute to the tourism industry through the reduction of shoreline erosion, retaining sand for beach replenishment to create a suitable environment for onshore resort tourism (Jackson et al., 2012; International Coral Reef Initiative, 2018).

### The next steps with restoration at the regional scale

While novel bio-enhancing habitat creation and other bottom-up conservation strategies (*i.e*., assisted evolution of coral species) are desperately needed on the world’s coral reefs, so are rapid and targeted microclimate management techniques. Chemical and thermal stress, coastal development, and unsustainable fishing have caused the loss of over 50% of global reefs to date (Gardner et al., 2005; Bruno & Selig, 2007; De’ath et al., 2012). Concerted efforts are underway worldwide to halt CO_2_ emissions (United Nations, 2017), and mounting evidence suggests that active interventions must be implemented to preserve, maintain, and enhance biodiversity and valuable ecosystem functions (Boström-Einarsson et al., 2020). To this end, the research team is developing top-down restoration technologies to manage marine microclimates better, creating valuable refuges for vulnerable species along threatened coastlines. These techniques include the strategic design deployment of habitats made from advanced material substrates that protect resident species from marine pathogens, extreme thermal events, local pollution, poor water quality, and changes in ocean chemistry. Theoretically, using both growth-enhancing Oceanite mineral substrates and select microclimate interventions together can provide healthy source populations and marine refuges along entire coastlines as we work as a global community to lower anthropogenic ocean stressors.

Future research will focus on the application of developed ARs for species-specific recruitment and growth rates of reef-building, calcifying species. We will also examine the regional application of ARs into marine spatial planning, with a specific focus on population connectivity, spillover effects, and assisted migration along marine corridors. Advanced materials increase the customization of ARs, creating a unique opportunity to simultaneously target biological, socio-economic, and cultural benefits across both public and private sectors, returning profit to coastal communities, empowering local people, and improving the scale of restoration success.

## Conclusions

It was demonstrated that nano-engineered ARs offer dynamic substrate orientations, structural complexity, and high-quality colonizable habitat that calcifying organisms settle on quickly (about a year) and attract fish from nearby habitats. Here, the AR can be tailored structurally and chemically to meet habitat-specific requirements for targeted biological communities. Initial results from these pilot studies suggest that when the ARs are deployed in MPAs and low-traffic areas, they have the potential to accumulate nearly 100% cover on all surfaces. Our research also found that the interior matrix of the Oceanite blocks was entirely colonized by cryptic benthic invertebrates (*e.g*., sponges, polychaetes, bivalves, ascidians, *etc*.), suggesting that the porous nature and complex microtopography of Oceanite facilitate biodiversity and settlement. This is a feature that is currently lacking in all other marine AR materials (*i.e*., metal, concrete, *etc*.), which are not tailored for the application. The results from our preliminary investigations of benthic communities on Oceanite mineral matrices also suggest that the structural design of future ARs should integrate the substrates angled to reduce sedimentation and create a variety of light exposure levels to enhance biodiversity. Our observations of fish abundance among sites suggest that deploying networks of large ARs with high vertical relief over broad spatial scales (*i.e*., sites with >30 m in diameter) may be critical to address the conservation goals related to increasing the abundance of ecologically and commercially important reef fish.

## Supplemental Information

10.7717/peerj.18487/supp-1 Supplemental Information 1 IntelliReefs Sint Maarten Data: Benthic Coverage, Fish Behaviour, Fish Abundance, Fish Community Composition.

## Additional Information and Declarations

### Data Availability

The following information was supplied regarding data availability: The benthic community percent cover, as well as fish abundance, diversity, and behavioural data are available in the Supplemental File.
